# The Effect of Reactive Electric Field-Assisted Sintering of MoS_2_/Bi_2_Te_3_ Heterostructure on the Phase Integrity of Bi_2_Te_3_ Matrix and the Thermoelectric Properties

**DOI:** 10.3390/ma15010053

**Published:** 2021-12-22

**Authors:** Yanan Wang, Cédric Bourgès, Ralph Rajamathi, C. Nethravathi, Michael Rajamathi, Takao Mori

**Affiliations:** 1 International Center for Materials Nanoarchitectonics (WPI-MANA), National Institute for Materials Science (NIMS), Namiki 1-1, Tsukuba 305-0044, Japan; s1936013@s.tsukuba.ac.jp (Y.W.); BOURGES.Cedric@nims.go.jp (C.B.); 2 Graduate School of Pure and Applied Sciences, Tsukuba University, Tennoudai 1-1-1, Tsukuba 305-8671, Japan; 3 Materials Research Group, Department of Chemistry, St. Joseph’s College, 36 Lalbagh Road, Bangalore 560027, India; ralphrajamathi@gmail.com (R.R.); mikerajamathi@rediffmail.com (M.R.); 4 Department of Chemistry, Mount Carmel College, 58 Vasanthnagar, Bangalore 560052, India

**Keywords:** thermoelectric, nanocomposite, Bi_2_Te_3_, MoS_2_, hydrothermal synthesis, reactive SPS

## Abstract

In this work, a series of Bi_2_Te_3_/X mol% MoS_2_ (X = 0, 25, 50, 75) bulk nanocomposites were prepared by hydrothermal reaction followed by reactive spark plasma sintering (SPS). X-ray diffraction analysis (XRD) indicates that the native nanopowders, comprising of Bi_2_Te_3_/MoS_2_ heterostructure, are highly reactive during the electric field-assisted sintering by SPS. The nano-sized MoS_2_ particles react with the Bi_2_Te_3_ plates matrix forming a mixed-anion compound, Bi_2_Te_2_S, at the interface between the nanoplates. The transport properties characterizations revealed a significant influence of the nanocomposite structure formation on the native electrical conductivity, Seebeck coefficient, and thermal conductivity of the initial Bi_2_Te_3_ matrix. As a result, enhanced *ZT* values have been obtained in Bi_2_Te_3_/25 mol% MoS_2_ over the temperature range of 300–475 K induced mainly by a significant increase in the electrical conductivity.

## 1. Introduction

Thermoelectric (TE) materials can convert heat to electricity or vice versa and the efficiency of the conversion is characterized by the dimensionless figure of merit *ZT*, *ZT* = *S*^2^σ*T*/κ, wherein the Seebeck coefficient (*S*), the electrical conductivity (*σ*), and the thermal conductivity (*κ*, including electronic component *κ_e_*, lattice component κ_l_, and bipolar component *κ_b_*) are three interdependent properties which depend on the absolute temperature (*T*) [1,2,3]. It represents that, at a given temperature, a ‘good’ TE material should be characterized simultaneously by a large value of power factor (*PF* = *S*^2^σ) and a low value of *κ*. However, the tight trade-off due to the interdependence between *S*, *σ*, and *κ* makes the enhancement of the *ZT* values challenging. In order to develop high-performance TE materials, several efficient concepts have been employed, such as nano structuring [4,5,6], doping [7,8], solid solutions [9], energy filtering [10], band convergence [11], and magnetic enhancement [12,13]. Recently, developing composites for tuning the TE characteristics materials appear promising and constitute a striking strategy to improve the performance of TE materials [14,15,16,17,18].

Among the state-of-the-art TE materials, Bi_2_Te_3_-based compounds are well known as the best materials for near room-temperature applications and the research on nanostructured Bi_2_Te_3_-based compounds is increasing [19,20,21,22,23,24,25,26]. As the most popular candidate for TE power generation and refrigeration [19], quintuple-layered Bi_2_Te_3_ is also known as a topological insulator (TI), with an insulating bulk and metallic surface states protected by time-reversal symmetry [27,28], meaning charge carriers are not backscattered by nonmagnetic impurities and defects.

Bi_2_Te_3_/MoS_2_ nanocomposites have exhibited attractive properties in the field of catalysis due to the conductive interfaces between Bi_2_Te_3_ and MoS_2_ [29]. MoS_2_, a graphene-analogue, is known for its reasonable TE performance [30,31,32,33] because of its physical properties [34], such as discretized density of states and high carrier mobilities. Based on these studies, exploring the TE properties of Bi_2_Te_3_/MoS_2_ appears to be interesting. To the best of our knowledge, until now, there are very few studies on this system. Keshavar et al. [35] fabricated *p*-type MoS_2_/(Bi_0.2_Sb_0.8_)_2_Te_3_ composites by a top-down approach and found that the addition of MoS_2_ nanoparticles can reduce the thermal conductivity due to additional scattering of phonons resulting in an enhanced *ZT* at temperatures higher than 370 K. More recently, Tang et al. [23] used a bottom-up method to prepare *n*-type MoS_2_/Bi_2_Te_3_ nanocomposites, which demonstrated a power factor of 1.83 mW m^−1^K^−2^ at ~319 K that is 30% higher than that of the pristine Bi_2_Te_3_. However, the thermal conductivity, as well as the *ZT* of the samples, were not reported in their work despite an expected positive influence of the nanocomposite interface to create some phonon barriers. Thus, systematic studies on Bi_2_Te_3_/MoS_2_ composites should go further. In Bi_2_Te_3_/MoS_2_ composites, Bi_2_Te_3_ is expected to act as a template that controls the growth and loading of MoS_2_ which connects the Bi_2_Te_3_ nanoplates to promote electron transfer and assist the formation of heterostructures with well-defined interfaces leading to enhanced phonon scattering. Moreover, the contact between the Bi_2_Te_3_ and the MoS_2_ phase would be a tunable interface to significantly enhance the electronic transport of the final nanocomposite materials.

In this study, layered Bi_2_Te_3_/MoS_2_ nanocomposites of varying compositions were synthesized through a hydrothermal reaction followed by the spark plasma sintering (SPS) process. The influence of MoS_2_ content combined with an atypical synthesis approach on the transport behavior of nanocomposites was explored.

## 2. Materials and Methods

### 2.1. Synthesis of Hexagonal Nanoplatelets of Bi_2_Te_3_ and Bi_2_Te_3_/MoS_2_ Heterostructures

Hexagonal nanoplatelets of Bi_2_Te_3_ and Bi_2_Te_3_/MoS_2_ heterostructures were synthesized as described previously [29]. Bi_2_Te_3_ hexagonal nanoplatelets were prepared by adding Bi_2_O_3_ (0.5515 g, 1.18 mmol), TeO_2_ (0.5745 g, 3.60 mmol), and 4 M NaOH solution (6 mL) into a solution of PVP (0.96 g) in ethylene glycol (42 mL). The mixed yellow suspension was stirred vigorously for 30 min and transferred into a 50 mL Teflon-lined autoclave and sealed in a stainless-steel canister. The autoclave was heated at 200 °C for 4 h. The grey colored product was washed by centrifugation several times using distilled water followed by acetone, and dried in air at room temperature.

Bi_2_Te_3_/MoS_2_ heterostructure with 75 mol% MoS_2_ was prepared as follows. The obtained hexagonal nanoplatelets of Bi_2_Te_3_ (0.205 g, 2.56 × 10^−4^ mol) was added into 45 mL water and stirred for 30 min to get a dispersion. Ammonium tetrathiomolybdate (0.2 g, equivalent to 7.68 × 10^−4^ mol of MoS_2_) was added to this dispersion following 15 min of stirring. Hydrazine hydrate (5 mL) was added to the mixture and the stirring continued for another 15 min. The mixture was then transferred into a Teflon-lined autoclave, sealed, and heated in a hot air oven at 200 °C for 24 h. The black solid obtained was separated by centrifugation, washed several times with distilled water followed by acetone, and dried under ambient conditions. Bi_2_Te_3_/X mol% MoS_2_ heterostructures with X = 0, 25, 50 were also synthesized. In all cases, the mass of ammonium tetrathiomolybdate used was kept constant (200 mg).

### 2.2. Synthesis of Bi_2_Te_3_/X mol%MoS_2_ Bulk Samples

The powders obtained by the hydrothermal synthesis were loaded into a graphite die (Φ 10 mm) and sintered by spark plasma sintering (Dr. Sinter, SPS-322Lx, Osaka, Japan) under a uniaxial pressure of 50 MPa. The sintering was performed in a partial argon atmosphere at 623 K for 5 min (heating and cooling rate of 100 K min^−1^). The sintered pellets were then cut and polished to the required shapes and dimensions for the different characterizations. All properties were measured on the same specimen along the in-plane axis perpendicular to the SPS uniaxial pressure. The densities measured by Archimedes’ method were 6.58, 6.28, 5.74, and 4.64, respectively, for X = 0, 25, 50, and 75 mol% MoS_2_.

### 2.3. Chemical and Structural Characterization

The phase compositions were characterized by powder X-ray diffraction (Rigaku, Ultima III, Tokyo, Japan) with Cu ${Κ}_{\alpha }\;$ radiation. Data were collected over a 2*θ* range of 10–90° with a step size of 0.02° and a scan rate of 3°/min. Microstructural and composition analysis of the samples were performed by a field-emission ultra-high resolution scanning electron microscope (SEM; SU4800 Hitachi) and a mini-SEM (TM3000, Hitachi, Tokyo, Japan) equipped with an energy-dispersive spectrometer (EDS).

### 2.4. Physical Property Measurements

The thermal diffusivity *α* and heat capacity *C_p_* were measured using LFA-467 Hyperflash (Netzsch, Burlington, MA, USA) under a flowing argon atmosphere (50 mL/min). The thermal conductivity *κ* was derived as a product of the sample’s density (measured by Archimedes’ method), thermal diffusivity, and heat capacity *C_p_*. The sintered disks were cut into rectangular bars for simultaneous electrical conductivity and Seebeck coefficient measurements using a commercial instrument (ZEM-2, ULVAC Shinku-Riko, Yokkaichi, Japan) with a standard four-probe configuration under a partial helium atmosphere. All property measurements were performed on the same specimen. Taking into account the strong preferred orientation of the layered structure, *S*, *ρ*, and *κ* measurements were all measured along a plane perpendicular to the SPS pressure direction, namely, ‘in-plane axis’. Hall Effect measurement were carried out using a physical properties measurement system (PPMS; Quantum Design, San Diego, CA, USA), in a magnetic field of −7 T to 7 T at 300 K.

## 3. Results and Discussion

### Microstructure and Chemical Composition

**Figure 1:**
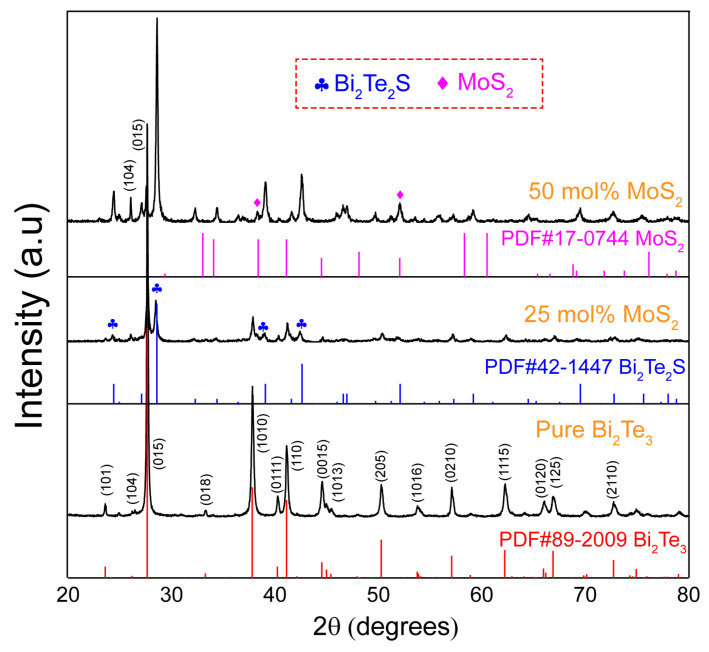
PXRD patterns of the Bi_2_Te_3_/X mol% MoS_2_ heterostructures (X = 0, 25, 50) after SPS.

**Figure 2:**
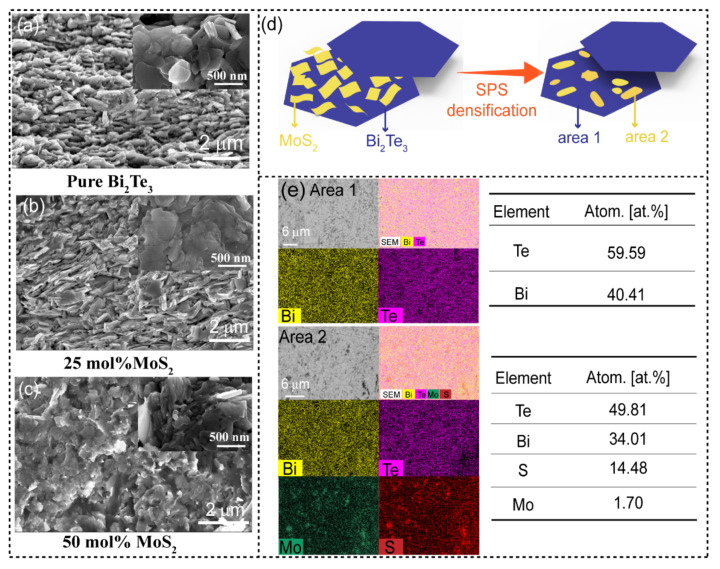
Fracture surface SEM images of Bi_2_Te_3_/X mol% MoS_2_ after spark plasma sintering with (**a**) X = 0, (**b**) X = 25, and (**c**) X = 50; the insets correspond to the higher magnification images of the fracture surfaces; (**d**) schematic depiction of Bi_2_Te_3_/MoS_2_ heterostructure before and after SPS; and (**e**) representative elemental mapping of two distinct areas of the X = 25 sample with their corresponding atomic compositions.

The powder X-ray diffraction (PXRD) patterns of the Bi_2_Te_3_/X mol%MoS_2_ (X = 0, 25, 50) nanocomposites after SPS and the typical native powder of Bi_2_Te_3_/75 mol%MoS_2_ are shown in Figure 1 and Figure S1, respectively. While the SEM image of the pristine Bi_2_Te_3_ (Figure S1b) shows hexagonal platelets with smooth surfaces, Bi_2_Te_3_/75 mol%MoS_2_ heterostructure (Figure S1c) shows the hexagons of Bi_2_Te_3_ with a rough surface due to the growth of MoS_2_ layers. The diffraction peaks of the pristine sample X = 0 are in good agreement with the standard data for Bi_2_Te_3_ (JCPDS no. 89-2009) and the phase purity is confirmed through the Rietveld refinement (Figure S2a and Table S1), which highlight low-reliability factors attesting to the non-degradation of the native powder during the sintering process at the select temperature (T = 623 K). However, despite the low sintering temperature, the presence of MoS_2_ nanoflake on the Bi_2_Te_3_ matrix induces the formation of the Bi_2_Te_2_S-tetradymite phases as visible in the X = 25 and 50 PXRD patterns (Figure 1) and further confirmed by pattern matching (Figure S2b,c), which suggests a reaction/degradation occurred during the sintering process. The tetradymite phase (Bi_2_Te_2_S) is likely obtained by the reaction of the metastable 1T-MoS_2_ nanoparticle with the Bi_2_Te_3_ main matrix surface, affecting the respective microstructure of the sample as further discussed in the next section with the scanning electron microscopy (SEM) images (Figure 2). In the native powder (X > 0), the surface of the Bi_2_Te_3_ nanoplatelets is uniformly covered with layers of metallic 1T-MoS_2_ [29], which is schematically depicted in Figure 2d and the corresponding typical SEM image is shown in Figure S1c. It is well known that the chemical reactivity of nanoparticles is enhanced on account of the far larger surface areas than similar masses of larger-scale materials. A combination of the enhanced reactivity and the high energy available during electric-field assisted sintering by SPS makes the surface ionic exchange between MoS_2_/Bi_2_Te_3_ nanoplatelets become propitious leading to the formation of tetradymite phase as well as off-stoichiometric MoS_2−x_ nanoplates and/or Mo_2_S_3_ phase at the interface [36]. It can be pointed out that there is a possibility of partial Mo doping in Bi_2_Te_3_ according to a recent theoretical report [37]. Besides, the role of the electric field-assisted sintering in the reactive densification is obvious considering the report of Bi_2_Te_3_-MoS_2_ composite realized by the hot-pressing (HP) method, wherein there was no reaction between the two phases [23]. However, further investigations are required to fully understand the mechanism of the reaction. It can be noticed that for the low content of MoS_2_ (25 mol%), the Bi_2_Te_3_ phase remained the main phase according to the intensities of the major peaks (Figure 1). In contrast, the Bi_2_Te_2_S dominates the matrix while the native Bi_2_Te_3_ main peaks are reduced significantly for the 50 mol% sample. The different phases have been confirmed by the SEM composition analysis using energy dispersive spectroscopy (EDS) (Figure 2e and Figures S3–S5) and the results are in line with the PXRD observation. The Bi_2_Te_3_/75 mol% MoS_2_ nanocomposite was investigated but the PXRD pattern (Figure S6) revealed a plethora of secondary phases that are difficult to identify with the conventional PXRD resolution revealing the limit in the MoS_2_/Bi_2_Te_3_ ratio, which can be mixed to be able to control a constructive nanocomposite formation. Considering the resulting poor transport properties (Figures S7 and S8), this composition has not been further developed in the study and discussion.

Figure 2a–c depicts the SEM images of freshly fractured surfaces of Bi_2_Te_3_/X mol% MoS_2_ (X = 0, 25, 50) samples which show the archetypal plate-shaped particles for the X = 0 and 25 samples as expected for these layered nanocomposite materials. The high-magnification image (Figure 2a, inset) shows that the pristine Bi_2_Te_3_ has an obvious typical hexagonal lamellar structure with a smooth surface and a preserved particle size ranging around ~500 nm. The shape of the native nanoplates of Bi_2_Te_3_ becomes ill-defined in the composite with 25 mol%MoS_2_ (Figure 2b, inset), and the plates do not present the characteristic rough surface that indicates the MoS_2_ presence on the surface of the Bi_2_Te_3_ platelets (cf. Figure S1c). This is in agreement with the merging/reaction of the MoS_2_ and platelets of Bi_2_Te_3_ to form the Bi_2_Te_2_S interface as observed through the PXRD analysis (Figure 1) and confirmed by EDS analysis (Figure 2e). Notably, the microstructure of the composite with 50 mol% MoS_2_ is far different from that of the 25 mol%. The shape of the crystals became blurry due to the extensive merging of nanoplates promoted by the high content of MoS_2_. 

To further understand the effect of the nanocomposite formation, the electrical and thermal transport properties of samples were characterized and compared in the in-plane direction. Due to the plate shape nature of these layered structures, the SPS will promote a certain degree of texturing along the ‘in-plane’ direction perpendicular to the SPS load axis. Thus, texturing will be favorable to a large charge carrier mobility as well as a longer relaxation time of the phonon scattering. Consequently, the electrical and thermal conductivity will be higher in the ‘in-plane’ axis as reported extensively in the literature [38,39,40].

**Figure 3:**
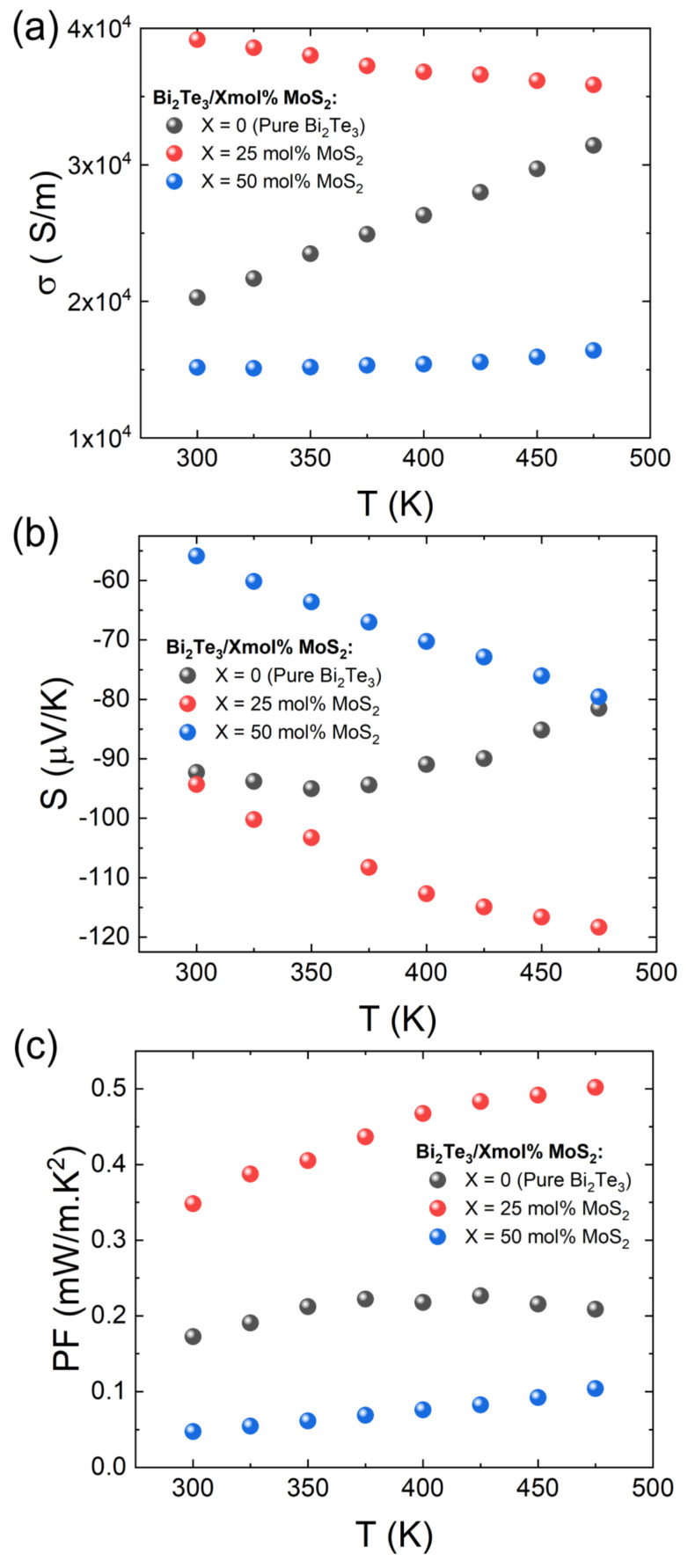
Temperature dependence of (**a**) electrical conductivity σ, (**b**) Seebeck coefficient *S*, and (**c**) power factor *PF* of the Bi_2_Te_3_/X mol%MoS_2_ (X = 0, 25, 50) nanocomposites.

As displayed in Figure 3, the sample of Bi_2_Te_3_ produced using hydrothermal method followed by SPS has a relatively low electrical conductivity caused by the reduction of crystal size and the relatively low sample density (about 85%), which induced much more scattering interfaces at the grain boundaries and/or the pores [41]. The resulting combination of both features will affect significantly the charge carrier mobility as it has been confirmed through the Hall effect measurement (Table 1) wherein the carrier mobility of the X = 0 sample is estimated with a low value of *μ_e_* = 10.90 cm^2^ V^−1^s^−1^. Additionally, it is observed that the Bi_2_Te_3_/0 mol%MoS_2_ is characterized by a non-degenerate *n*-type semiconducting behavior (positive dσ/dT) with T increasing (Figure 3a), likely due to a slight Te-rich composition (Figure S3) and a moderate carrier concentration in the ≈10^19^ cm^−3^ range (Table 1) [42]. Compared with the Bi_2_Te_3_/0 mol%MoS_2_ sample, the nanocomposite formation in the Bi_2_Te_3_/25 mol%MoS_2_ sample induces a constructive effect leading to a substantial enhancement of the electrical conductivity, especially in the room temperature range. It is interpreted that the formation of Bi_2_Te_2_S between the nanoplates leads to a superior electrical contact by comparison with the Bi_2_Te_3_ sample wherein the nanoplates are not fully connected (Figure 2a,b). Consequently, the carrier mobility is improved, mainly in the out-of-plane axis, and therefore promoting an overall higher electrical conductivity. It is sustained experimentally by the Hall effect measurement, which highlighted a largely improved mobility in the X = 25 sample with a five times improved carrier mobility up to *μ_e_* = 51.50 cm^2^ V^−1^s^−1^ (Table 1). However, the *σ* of the nanocomposite sample with 50 mol% MoS_2_ is drastically reduced in consistence with the dominant presence of the tetradymite phase (Figure 1) which reduced the overall carrier concentration of the nanocomposite. Indeed, the tetradymite phase is known to have a wider bandgap (*E_g_* ≈ 0.3 eV) and a lower electrical conductivity than Bi_2_Te_3_ [43,44]. In addition, the influence of the fractured microstructure (Figure 2c) cannot be ruled out, which could be the major contribution of the large reduction of the *σ* in the Bi_2_Te_3_/75 mol%MoS_2_ composite by reducing the carrier mobility.

The Seebeck coefficient (*S*) behavior has been investigated as an effective indicator of prevalent carrier type as well as the effect of nanocomposite formation on the electrical transport properties. As shown in Figure 3b, all the samples show a negative *S* indicating that the predominant carriers are the electrons (*n*-type). The Seebeck coefficient in the Bi_2_Te_3_/25 mol% MoS_2_ sample is comparable with the pristine sample in the near room temperature range, agreeing with the fact that Bi_2_Te_3_ is the main phase in Bi_2_Te_3_/25 mol% MoS_2_ composite. The *S* values do not saturate over the whole temperature range likely due to the proficient influence of the tetradymite minor phase. The *S_max_* (−118.3 μV K^−1^ at 475 K) is about 1.47 times larger than the pristine Bi_2_Te_3_ (−81 μV K^−1^ at 475 K). This larger |*S*| is in consistence with the reduced carrier concentration compared with the pristine Bi_2_Te_3_ sample (Table 1). Further increase in mol% of MoS_2_ dilapidates the value of |*S*| to around 40% lower in the Bi_2_Te_3_/50 mol% MoS_2_ sample. Contrary to the electrical transport tendency moving to a ‘semiconductor-like’ behavior with increasing MoS_2_ molar ratio, the decreasing of |*S*| suggests a more ‘metal-like’ behavior that is not representative of the tetradymite main phase, which will be represented by a higher |*S*| (≈−190 μV K^−1^ at 300 K) [43,44]. However, this Seebeck value appears consistent with the report of the MoS_2_/Mo_2_S_3_ nanocomposite [45], which gives us an insight into the non-negligible role of the S-deficient MoS_2_ phases observed in the EDS mapping analysis (Figures S4 and S5). Therefore, further investigations with higher accuracy than PXRD and EDS are required to fully confirm the presence of this latter phase and its contribution to the nanocomposites’ transport properties. Based the things considered above, the 25 mol% MoS_2_ content is the optimum value to massively improve the electrical conductivity as well as preserve a large Seebeck coefficient in this typical bulk synthesis approach. Ultimately, the highest power factor (S^2^σ) at room temperature was obtained for the 25 mol% MoS_2_ sample with *PF* = 0.35 mW m^−1^K^−2^ at 300 K that increases to 0.5 mW m^−1^K^−2^ at 475 K, as shown in Figure 3c.

The thermal properties of the nanocomposites were characterized to probe the influence of the nanocomposite formation on the thermal transport behavior. The thermal conductivity *κ* is expressed by *κ = κ_e_* + *κ_l_* + *κ_b_*. The *κ_e_* can be estimated from the Wiedemann–Franz law:

*κ*_*e*_ = *L* × *T* × *σ*, (1)

where *L* is the Lorentz number, calculated by the equation:

*L* = 1.5 + exp[−|S|/116] (2)

**Figure 4:**
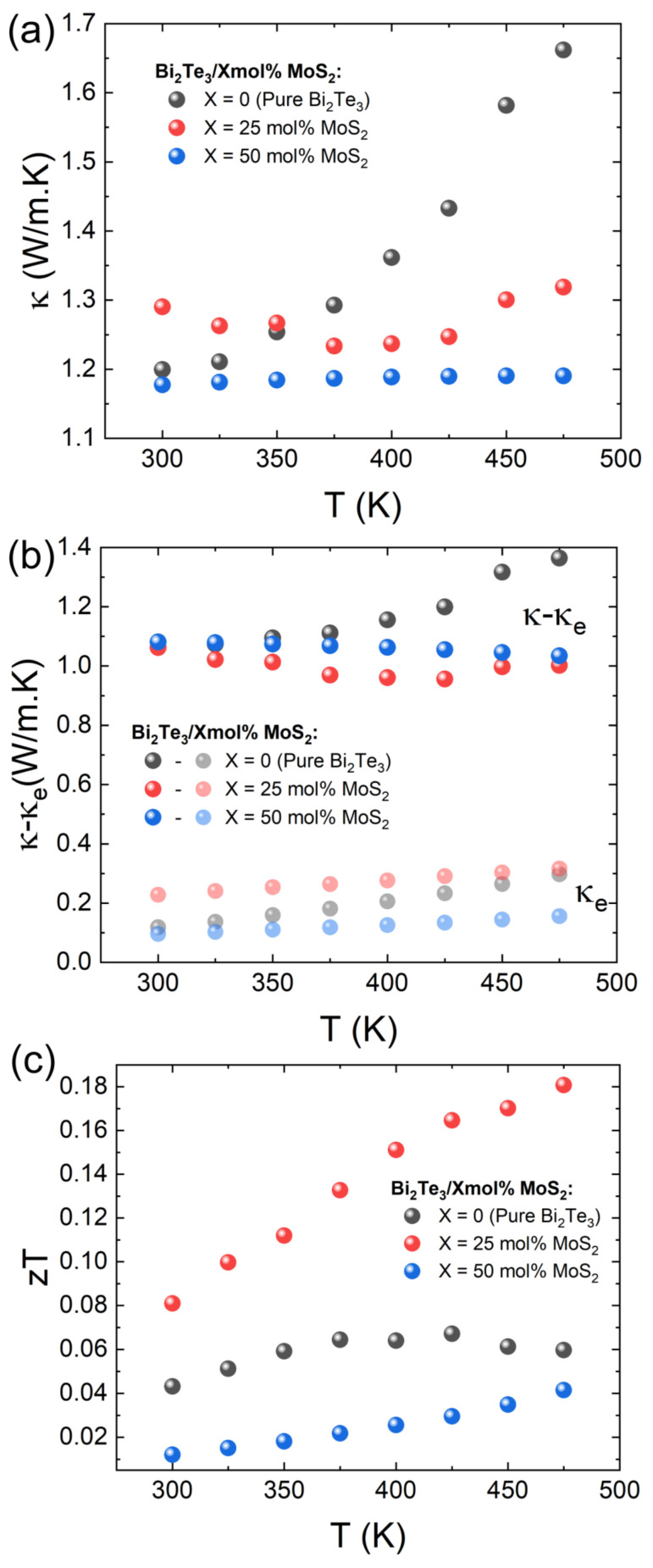
Temperature dependence of (**a**) total thermal conductivity *κ*; (**b**) *κ*-*κ_e_*; and (**c**) *ZT* of Bi_2_Te_3_/X mol%MoS_2_.

The temperature dependence of *κ* and *κ*-*κ_e_* are shown in Figure 4a,b, respectively. The average thermal conductivities of ~1.37, 1.27, and 1.19 W m^−1^ K^−1^ were correspondingly determined for 0 mol%, 25 mol%, and 50 mol% MoS_2_ composites. It should be noted that the *κ* of the pristine sample (1.1 W/m.K at 300 K) is in the lower range of values reported for Bi_2_Te_3_, likely due to the relatively low density of the sample, which leads to an enhanced effective phonon scattering by the pores. The bipolar thermal conductivity at high temperatures (sharp upturn on both *κ* and *κ*-*κ_e_*) is marked in pristine Bi_2_Te_3_ and becomes negligible with the formation of the nanocomposite [46]. Interestingly, the decrease in the bipolar contribution in the Bi_2_Te_3_/MoS_2_ nanocomposite appears to be a consequence of interface creation between the Bi_2_Te_3_ matrix and the tetradymite Bi_2_Te_2_S formation, where the energy barrier was produced because of the mismatched bandgap. Thus, the minor carriers will be scattered preferentially and consequently suppress the *κ_b_* [47]. As revealed in Figure 4b, the *κ_l_* predominates in the heat transport process in the Bi_2_Te_3_/X mol% MoS_2_ nanocomposites. Except in the pristine Bi_2_Te_3_, the *κ_l_* of nanocomposites (X = 25 and 50) has a negative temperature dependence. Moreover, the interfaces produced by the different phases in composites play the role of a phonon scattering center and thus slightly decreases the *κ*. Besides, the Bi_2_Te_2_S, reported with an intrinsic low thermal transport thanks to the mixed anion occupancy, plays a non-negligible role in enhancing phonon scattering and therefore suppresses the *κ_l_*, which is commonly related to a structural distortion induced by the bonding heterogeneity induced by the mixed anion occupancy in this compound [48,49]. Therefore, compositing MoS_2_ in Bi_2_Te_3_ matrix not only effectively decreases the thermal conductivity but also suppresses the bipolar conduction *κ_b_*.

The temperature-dependent *ZT* values of the nanocomposites are plotted in Figure 4c. It is revealed that the Bi_2_Te_2_S formation (X = 25) has a beneficial effect to enhance the global *ZT* of the Bi_2_Te_3_/MoS_2_ nanocomposite, mainly due to the significant improvement of the electrical conductivity. However, the constructive effect is limited to a narrow MoS_2_ loading. The promotion of the tetradymite as the main phase and the S-deficient MoS_2_ byproduct rise conflicting transport mechanisms compared with the mechanisms in the nanocomposites of X = 0, 50, and 75, thus, the lower ZT values are observed for composites with X = 0, 50, and 75 (Figure 4c and Figure S8).

## 4. Conclusions

Novel *n*-type Bi_2_Te_3_/X mol% MoS_2_ nanocomposites were prepared by hydrothermal reaction combined with reactive electric field-assisted sintering. This unconventional approach led to the formation of additional phases in the nanocomposite samples due to the reaction between Bi_2_Te_3_ and MoS_2_. The optimum TE properties of the nanocomposites were obtained for the low MoS_2_ content of 25 mol% due to the interplanar contact improvement, produced by the tetradymite (Bi_2_Te_2_S) phase formation, which led to a substantial electrical conductivity improvement without affecting the Seebeck coefficient. As a result, the Bi_2_Te_3_/25 mol% MoS_2_ gives an enhanced ZT of 0.18 at 475 K, which is three times higher than the reference nanostructured Bi_2_Te_3_. This atypical approach gives an insight for further enhancement in the TE performance of nanoscale material by using constructive composite interface engineering.

## Figures and Tables

**Table 1:** Carrier concentrations and mobility of the Bi_2_Te_3_/X mol MoS_2_ nanocomposites.

Bi_2_Te_3_/X mol MoS_2_ Nanocomposite—Hall Effect at 300 K			
	**X = 0**	**X = 25**	**X = 50**
***n* (cm^−3^)**	8.94 × 10^19^	4.75 × 10^19^	3.20 × 10^19^
***μ_e_* (cm^2^ V^−1^s^−1^)**	10.90	51.50	29.58

## Data Availability

The data presented in this research study are available in this article.

## Supplementary Materials

The following are available online at https://www.mdpi.com/article/10.3390/ma15010053/s1, Figure S1: XRD pattern (a) and SEM image of the precursor powder of pristine Bi_2_Te_3_ (b) and Bi_2_Te_3_−MoS_2_ (25:75) heterostructure (c), Figure S2: Refinement of the PXRD pattern of the Bi_2_Te_3_/X mol% MoS_2_ nanocomposite for (a) X = 0, (b) X = 25, (c) X = 50, and (d) schematic structural representation of the Bi_2_Te_3_, Bi_2_Te_2_S, and 2H-MoS_2_, Table S1: Cell parameters, reliability factors, and atomic coordination obtained from Rietveld refinement of PXRD patterns of the pure Bi_2_Te_3_, Figure S3: The elemental mapping, EDS spectrum, and composition of pure Bi_2_Te_3_ (x = 0) sample, Figure S4: Line analysis of the chunk and the elemental mapping of Bi_2_Te_3_/25 mol% MoS_2_ sample, Figure S5: (a) The elemental mapping, EDS spectrum, and composition of Bi_2_Te_3_/50 mol% MoS_2_ sample as well as the line analysis of black chunk in Bi_2_Te_3_/50 mol% MoS_2_ sample, Figure S6: PXRD pattern of the Bi_2_Te_3_/75 mol% MoS_2_ nanocomposite, Figure S7: Temperature dependence of electrical conductivity *σ*, Seebeck coefficient *S* and power factor *PF* of the Bi_2_Te_3_/75% mol%MoS_2_ nanocomposite after spark plasma sintering, Figure S8: Temperature dependence of thermal conductivity *κ* and figure of merit *ZT* of the Bi_2_Te_3_/75% mol%MoS_2_ nanocomposite.
