# Supplementary material for: The Effect of Reactive Electric Field-Assisted Sintering of MoS2/Bi2Te3 Heterostructure on the Phase Integrity of Bi2Te3 Matrix and the Thermoelectric Properties
Source: Materials (Basel). 2021 Dec 22;15(1):53. doi: 10.3390/ma15010053 (PMC8746225; doi:10.3390/ma15010053)
Supplement: Supplementary file 1 [file materials-15-00053-s001.zip › materials-1469721-supplementary.pdf]

Supplementary Material

The Effect of Reactive Electric Field-Assisted Sintering of MoS_2/Bi_2Te_3 Heterostructure on the Phase Integrity of Bi_2Te_3 Matrix and the Thermoelectric Properties

Yanan Wang [1,2,†], Cédric Bourgès [1,†], Ralph Rajamathi [3], C. Nethravathi [3,4,*], Michael Rajamathi [3] and Takao Mori [1,2,*]

1 International Center for Materials Nanoarchitectonics (WPI-MANA), National Institute for Materials Science (NIMS), Namiki 1-1, Tsukuba 305-0044, Japan; s1936013@s.tsukuba.ac.jp (Y.W.); BOURGES.Cedric@nims.go.jp (C.B.)
2 Graduate School of Pure and Applied Sciences, Tsukuba University, Tennoudai 1-1-1, Tsukuba 305-8671, Japan
3 Materials Research Group, Department of Chemistry, St. Joseph's College, 36 Lalbagh Road, Bangalore 560027, India; ralphrajamathi@gmail.com (R.R.); mikerajamathi@rediffmail.com (M.R.)
4 Department of Chemistry, Mount Carmel College, 58 Vasanthnagar, Bangalore 560052, India
* Correspondence: nethravathic@gmail.com (C.N.); MORI.Takao@nims.go.jp (T.M.)
† Equal contributions.

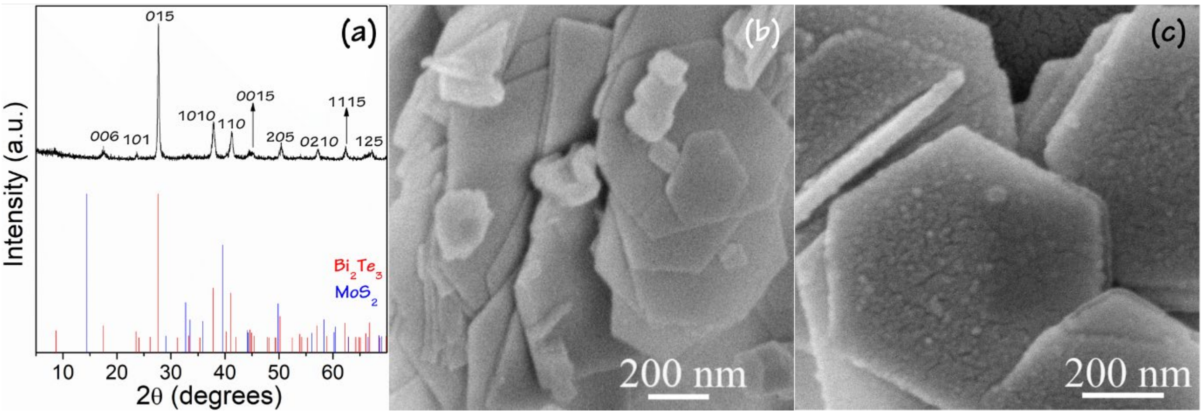

Figure S1. (**a**) XRD pattern of Bi_2Te_3–MoS_2 (75:25) heterostructure in comparison with expected peak positions of Bi_2Te_3 and MoS_2; SEM images of (**b**) pristine Bi_2Te_3 and (**c**) Bi_2Te_3–MoS_2 (25:75) heterostructure.

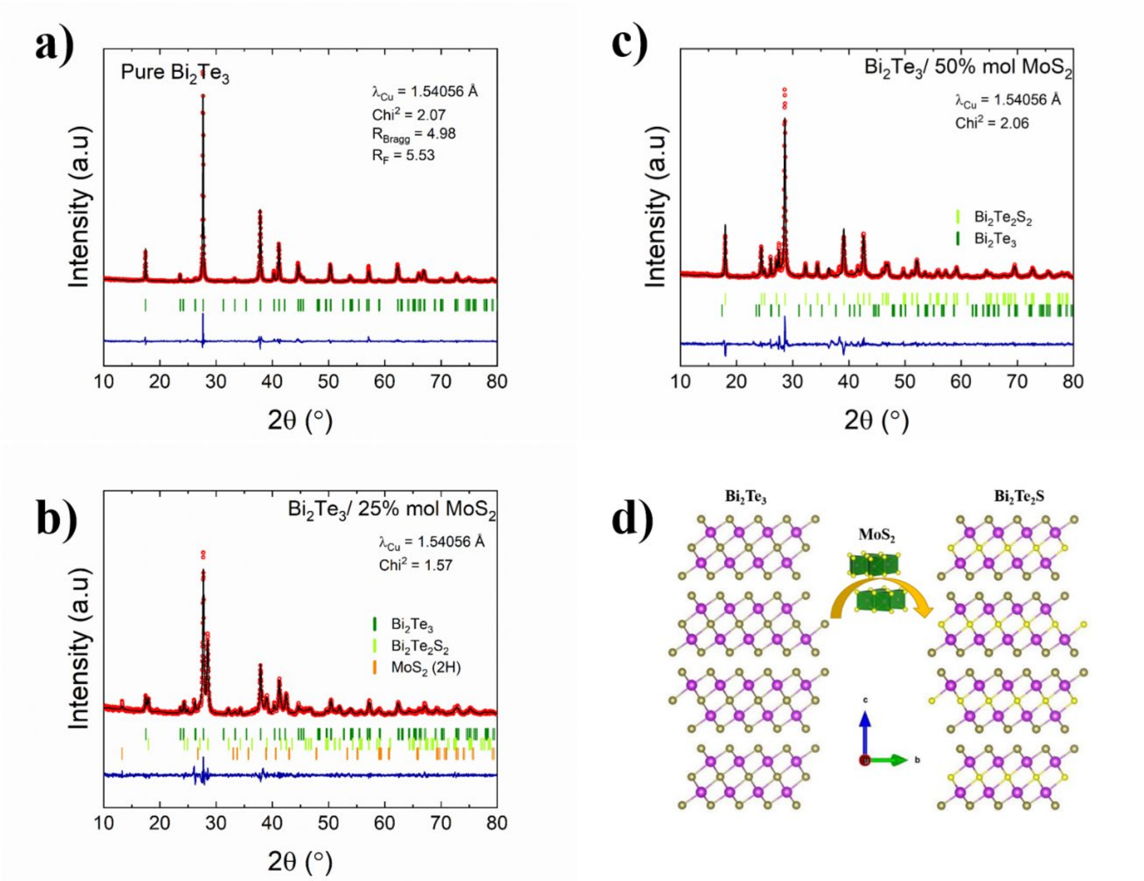

Figure S2. Refinement of the PXRD pattern of the Bi_2Te_3/X mol% MoS_2 nanocomposite for **a**) X = 0, **b**) X = 25, **c**) X = 50, and **d**) Schematic structural representation of the Bi_2Te_3, Bi_2Te_2S, and 2H-MoS_2.

Table S1. Cell parameters, reliability factors, and atomic coordination obtained from Rietveld refinement of PXRD patterns of the pure Bi_2Te_3.

**Pure Bi_2Te_3; $R\overline{3}m$; λ_{Cu} = 1.54056 Å; 300 K**					
a (Å)	c (Å)	V (Å)	Chi^2	R_{Bragg}	R_F
4.3851(1)	30.4791(1)	507.6(1)	2.07	4.98	5.53
Atom	x	y	z	Biso	Occ.
Bi	0	0	0.4005(1)	1.564(80)	1
Te1	0	0	0	0.891(197)	1
Te2	0	0	0.2088(1)	0.589(172)	1

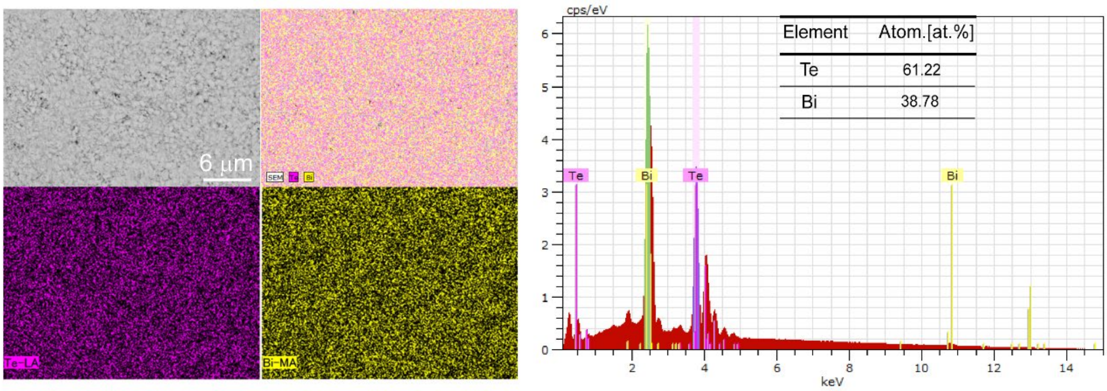

Figure S3. The elemental mapping, EDS spectrum and composition of pure Bi_2Te_3 (x = 0) sample.

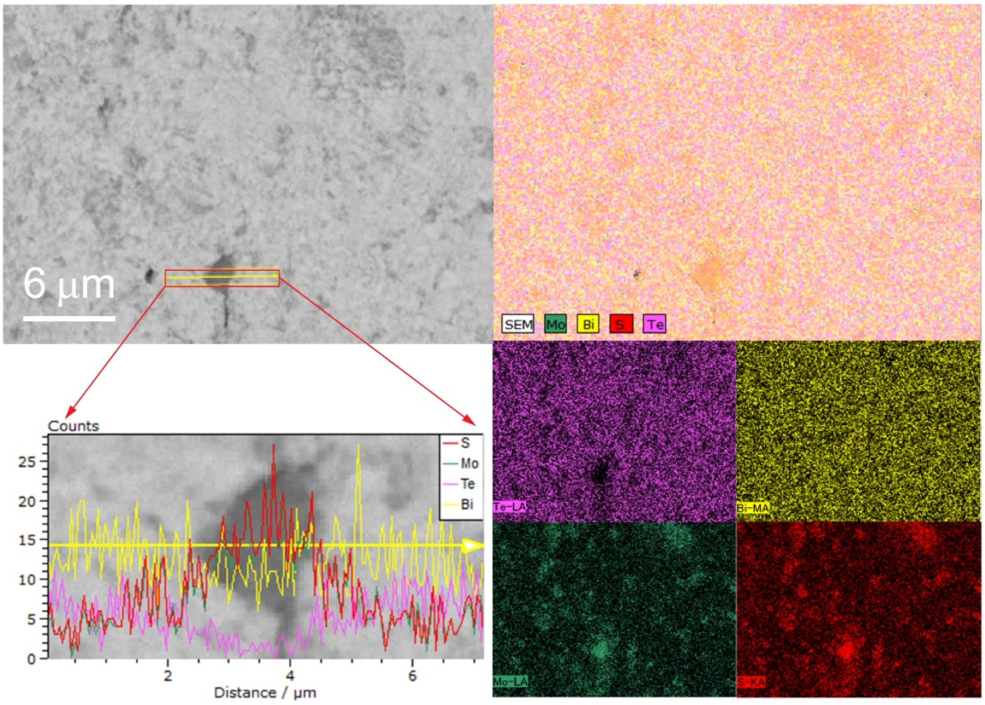

Figure S4. Line analysis of the chunk and the elemental mapping of Bi_2Te_3/25 mol% MoS_2 sample.

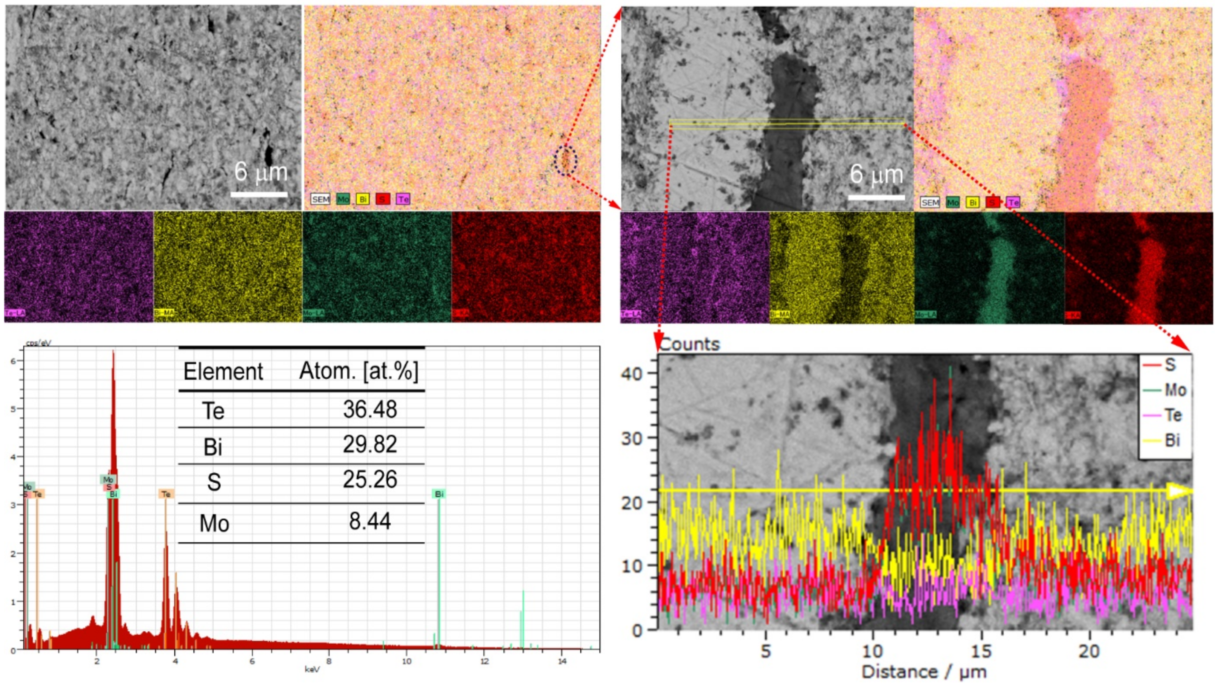

Figure S5. The elemental mapping, EDS spectrum and composition of Bi_2Te_3/50 mol% MoS_2 sample as well as the line analysis of black chunk in Bi_2Te_3/50 mol% MoS_2 sample.

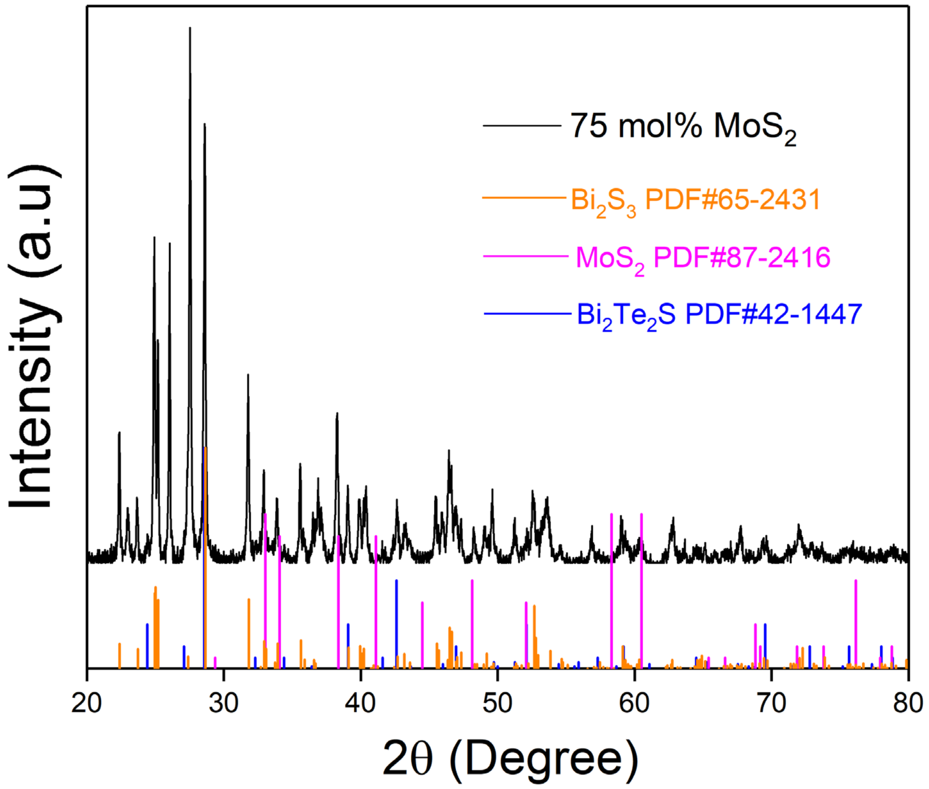

Figure S6. PXRD pattern of the Bi_2Te_3/75 mol% MoS_2 nanocomposite.

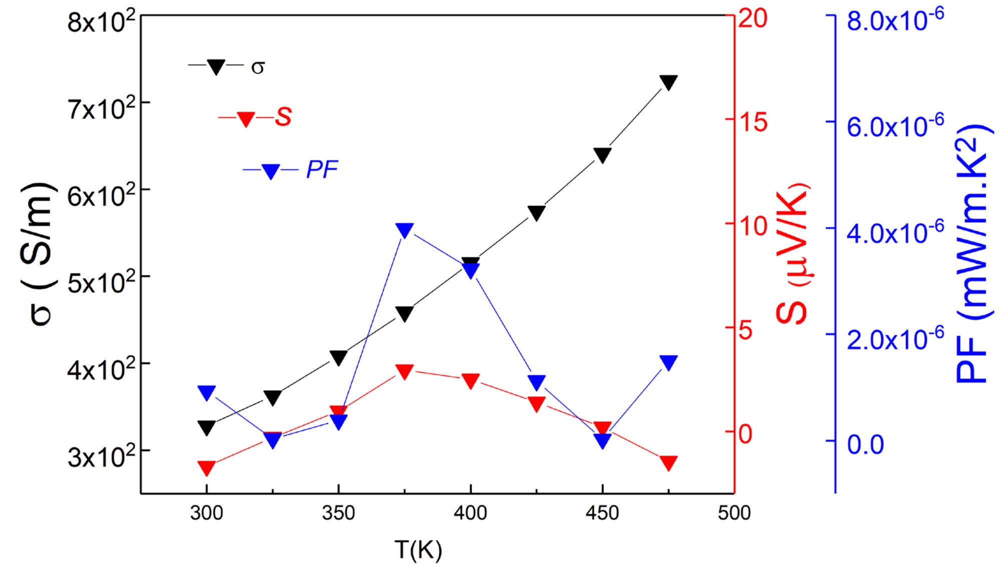

Figure S7. Temperature dependence of electrical conductivity *σ*, Seebeck coefficient *S* and power factor *PF* of the Bi_2Te_3/75% mol%MoS_2 nanocomposite after Spark Plasma Sintering.

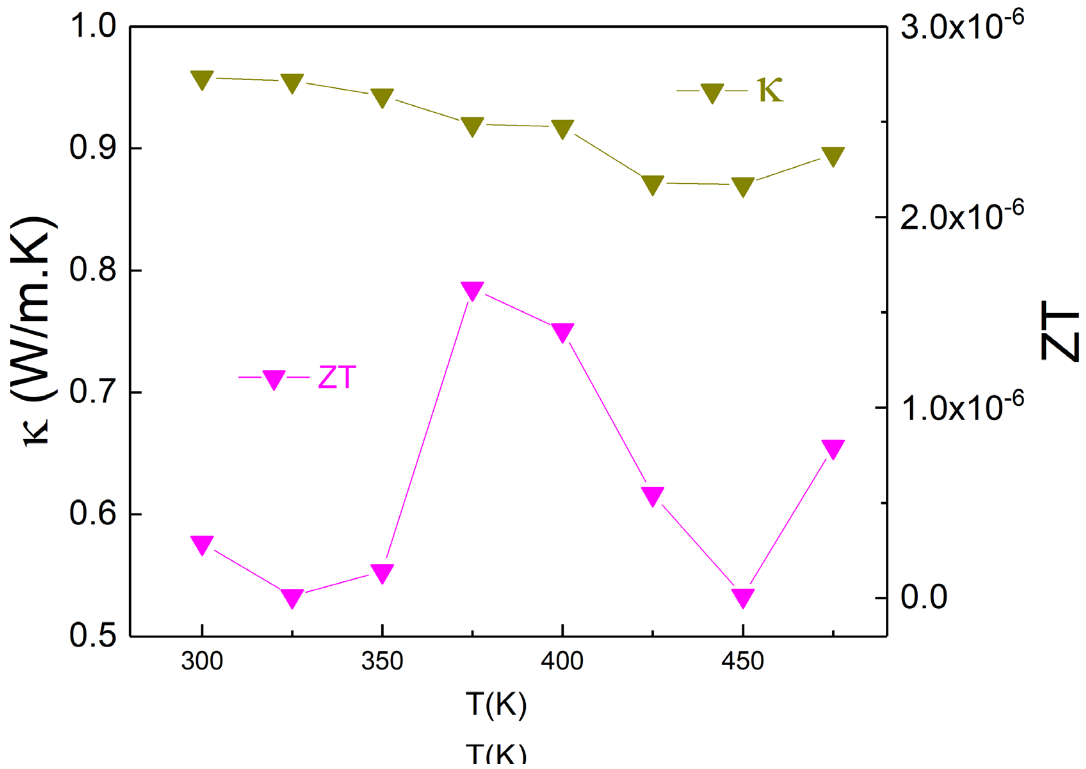

Figure S8. Temperature dependence of thermal conductivity *κ* and figure of merit *ZT* of the Bi_2Te_3/75% mol%MoS_2 nano-composite.
